# Spoonful of honey or a gallon of vinegar? A conditional COVID-19 vaccination policy for front-line healthcare workers

**DOI:** 10.1136/medethics-2020-107175

**Published:** 2021-05-11

**Authors:** Owen M Bradfield, Alberto Giubilini

**Affiliations:** 1 Melbourne School of Population and Global Health, The University of Melbourne, Melbourne, Victoria, Australia; 2 Oxford Uehiro Centre for Practical Ethics & Wellcome Centre for Ethics and the Humanities, University of Oxford, Oxford, UK; 3 University of Oxford, Oxford, UK

**Keywords:** COVID-19, health workforce, public health ethics, clinical ethics, right to refuse treatment

## Abstract

Seven COVID-19 vaccines are now being distributed and administered around the world (figure correct at the time of submission), with more on the horizon. It is widely accepted that healthcare workers should have high priority. However, questions have been raised about what we ought to do if members of priority groups refuse vaccination. Using the case of influenza vaccination as a comparison, we know that coercive approaches to vaccination uptake effectively increase vaccination rates among healthcare workers and reduce patient morbidity if properly implemented. Using the principle of least restrictive alternative, we have developed an intervention ladder for COVID-19 vaccination policies among healthcare workers. We argue that healthcare workers refusing vaccination without a medical reason should be temporarily redeployed and, if their refusal persists after the redeployment period, eventually suspended, in order to reduce the risk to their colleagues and patients. This ‘conditional’ policy is a compromise between entirely voluntary or entirely mandatory policies for healthcare workers, and is consistent with healthcare workers’ established professional, legal and ethical obligations to their patients and to society at large.

## Introduction

Currently, seven COVID-19 vaccines are being rolled out globally. Six hundred and forty-nine million doses have been administered globally, at a rate of 16.5 million doses per day (figures correct at the time of submission).^1^ As demand initially outweighs supply, vulnerable groups and front-line healthcare workers (FHCWs) are receiving priority access.^2^ FHCWs include healthcare staff who interact directly with patients, including doctors, nurses, allied health clinicians, pathology staff, security, cleaners and students. FHCWs provide essential services in settings that create a high risk of infection and transmission.^3^ Thus, an important ethical question is whether vaccination of FHCWs should be mandatory, as is already the case for influenza immunisation in some US jurisdictions and healthcare facilities. If so, how should this be enforced, and how should the personal preferences and autonomy of FHCWs be balanced against their professional responsibilities to act in the interests of patients and the public? This paper will define and defend a mildly coercive ‘conditional’ vaccination policy for FHCWs that represents a middle ground between an entirely voluntary and entirely mandatory approach. In our view, this strikes the best balance between the various ethical principles at stake.

## Justifying mandatory vaccination

Most public health interventions entail a trade-off between public benefits and private interests. Measures to promote public health and safety invariably create costs, which may be individual (infringement of autonomy), collective (social or economic costs) or often both. In order to justify costs incurred, coercive public health measures should aim to regulate serious risks, not merely ‘speculative, theoretical or remote’ risks.^4^ In this paper, we adopt Wertheimer’s definition according to which ‘coercion’ involves the threat of penalty for non-compliance and a degree of infringement of individual autonomy that leaves the individual with ‘no reasonable choice’ or ‘no reasonable alternative’.^5^ Hence, for the purpose of this paper, a ‘mandatory’ vaccination policy is considered coercive to the extent that the choice not to vaccinate is rendered difficult or is practically unavailable by the imposition of penalties. Such a policy may take various forms, as outlined below.

The COVID-19 pandemic poses a serious public health threat, as measured by mortality rate, incidence and prevalence. In just over a year, it infected over 131 million people and caused more than 2.8 million deaths.^6^ It disproportionately affects older people. Rates of hospitalisation and death are less than 0.1% in young children, but increase to more than 10% in people aged over 70 years.^7^ It also disproportionately affects racial and ethnic minorities in terms of frequency and severity of infection.^8^ In healthcare settings, the risks and impacts of COVID-19 are substantial.^9^ FHCWs are at high risk of infection and death,^10^ particularly older FHCWs and those who belong to racial and ethnic minority groups.^11^ FHCWs can transmit the virus to their patients, colleagues, families and the wider community.^12 13^ Moreover, hospital patients are generally sicker,^14^ which increases their risk of serious sequelae or death from COVID-19.^15^ When people avoid attending healthcare services for fear of contracting COVID-19, morbidity and mortality from other medical conditions, such as cancer, can increase.^16^ Therefore, there is a pressing need to reduce COVID-19 transmission specifically in healthcare settings and to ensure that patients and FHCWs, particularly those most at risk, do not continue to be disproportionately impacted by COVID-19. Any policy that increases vaccination rates among FHCWs is likely to prevent infection, morbidity and mortality, but needs to ensure equitable access and administration to FHCWs at greatest risk, such as older FHCWs and those from racial and ethnic minority backgrounds.

Even if a public health risk is serious, interventions aimed at reducing or preventing those risks must be shown to be effective before their implementation can be morally justified. However, with data yet to prove the effectiveness of a COVID-19 vaccine in healthcare settings specifically, we need to look to analogous situations for guidance. Studies demonstrate that influenza-related illnesses^17^ and deaths^18^ among elderly inpatients can be significantly reduced when just half of FHCWs are vaccinated against influenza. Mandatory influenza vaccination has been shown to be the most clinically effective^19^ and cost-effective^20^ strategy for increasing vaccination rates among FHCWs. Given the greater threat posed by COVID-19 than influenza in terms of infectivity and mortality, a mandatory COVID-19 vaccination policy could result in substantial benefits in healthcare settings. In fact, early data suggest that widespread and effective vaccination among FHCWs provides a safe hospital working environment, even in the presence of a high rate of SARS-CoV-2 infection in the community.^21^

The benefits of an effective mandatory COVID-19 vaccination policy for FHCWs must be balanced against the cost to FHCWs. No vaccine is 100% safe. Assuming that any approved COVID-19 vaccine turns out to be no less safe than existing vaccines in routine use, most adverse reactions would likely be relatively minor, except for exceedingly rare complications like vasculitis, encephalomyelitis, neuritis and paralysis (Guillain-Barré syndrome).^22^ However, some FHCWs may be at risk of severe adverse vaccine reactions. For instance, the UK government previously advised that FHCWs with a significant history of allergic reactions defer receiving the Pfizer-BioNTech COVID-19 vaccine after two FHCWs reported adverse reactions.^23^

Another cost of mandatory vaccination is that it might undermine goodwill between FHCWs and their employers. Overly coercive regulation may engender resentment,^24^ opposition^25^ and mistrust. FHCWs already experience higher rates of depression, anxiety, insomnia, post-traumatic stress disorder and burn-out from dealing with the tragic reality of this pandemic.^26^ Any mandate needs to be implemented in a way that is respectful and cognisant of the impact that the pandemic is having on the overwhelming majority of FHCWs, most of whom would likely consent to COVID-19 vaccination.

A mandatory vaccination policy also needs to address the cultural and informational needs of diverse racial and ethnic minority groups that have been disproportionately impacted by COVID-19,^27^ particularly since they are also over-represented in front-line healthcare work^28^ and would disproportionately bear the brunt of any vaccine mandate. Adequate resources need to be invested in information and trust campaigns that engage with affected communities and that work with specific cultural or religious views and concerns. To minimise any burdens associated with mandatory vaccination, all FHCWs must be provided with timely, accurate, comprehensible, culturally sensitive and balanced information about the benefits and risks, including areas of uncertainty. In addition, the vaccine must be accessible, offered at very little or no cost and accompanied by robust vaccine injury compensation mechanisms that cover medical expenses arising from vaccine complications.^29^

In most situations, the collective societal benefits of mandatory vaccination will outweigh these small risks of harm. However, individuals should not be required to accept serious existential risks for the sake of public safety. From an ethical perspective, what matters is that the collective benefits outweigh the risks, and that there is some limit to the kind of risks that can be imposed for the sake of those collective benefits. Even if a great collective benefit could be achieved, many would not consider it permissible to impose coercive measures that entail very large individual costs.^30^ For example, if an FHCW has a medical condition that significantly increases the likelihood of a serious adverse vaccine reaction, then it is morally permissible to exempt them from mandatory vaccination policies.

As Flanigan points out, it is permissible for an individual to expose others to risks of harm in order to defend oneself.^31^ By analogy, it may be permissible for an individual to expose others to risks of harm from infectious disease in order to defend oneself from serious vaccine reactions. However, the right to ‘self-defence’ is not absolute and whether or not an exemption on medical grounds is permitted will depend on the seriousness of the risk of harm to the FHCW. It is difficult to be prescriptive about the type of medical conditions that might authorise a medical exemption until we know more about the immediate and longer term effects of approved COVID-19 vaccines. Giubilini and Savulescu argue that the justification for coercive public health policies is stronger when individuals have a pre-existing moral obligation to fulfil those requirements and when such requirements are not overly demanding.^32^ Using other vaccines as an example, we believe that there are compelling ethical reasons to allow medical exemptions within a mandatory COVID-19 vaccination framework.

In view of the risks of vaccination, we need to explore alternatives. Without a vaccine, the use of hand hygiene, personal protective equipment (including face masks, eye goggles, protective suits and face shields), physical distancing, regular surveillance testing and quarantining are our best infection control strategies. In most hospitals, these procedures are already mandatory and FHCWs found to breach infection control guidelines face sanctions, such as disciplinary action or termination. Therefore, while mandatory vaccination may be more burdensome than hand washing, it may be no more burdensome than wearing full personal protective equipment for 12 hours/day. Prolonged use of personal protective equipment can cause physical problems, including breathing difficulties, pain, discomfort and dermatological reactions.^33^ Reducing face-to-face clinical contact through telehealth services can also reduce COVID-19 transmission.^34^ However, telehealth services cannot safely and effectively replace all face-to-face healthcare.^35^ So, while other measures exist to control the spread of COVID-19, they are likely inferior to vaccination and not without their own problems. They are, at best, adjuncts to an effective vaccine, but not a replacement. While some countries have achieved short-term elimination,^36^ this seems an unlikely long-term global goal, with many experts suggesting that COVID-19 will likely become endemic like seasonable influenza.^37^

Hence, assuming that, as is the case with influenza vaccination, mandatory policies are the most effective way of ensuring the highest vaccine uptake possible among FHCWs, mandatory COVID-19 vaccination of FHCWs appears morally justifiable at least from a utilitarian perspective, because it minimises risks and maximises patients’ and FHCWs’ welfare. The threat posed by COVID-19 is serious, the alternatives available are not a replacement for vaccination, less restrictive policies entail the risk of lower uptake and the public health benefits seem to outweigh the individual risks, except in limited cases where FHCWs are eligible for medical exemptions.

## A mild form of mandatory vaccination

Powers that promote public health may permissibly supersede individual liberty.^38^ For example, policies that maximise the number of vaccinated FHCWs promote public health by reducing COVID-19 transmission but deprive FHCWs of free choice and autonomy. The principle of the least restrictive alternative may balance these competing interests.^39^ It states that if two public health interventions both effectively address a pressing public health issue, then the least restrictive intervention should be preferred.^40^ The Nuffield Council on Bioethics has articulated an ‘intervention ladder’^41^ that grades public health policies according to the degree to which they restrict individual autonomy. Less intrusive measures must first be shown to be ineffective before more intrusive measures can be considered. In addition, more restrictive policies will generally achieve greater utility.^42^

At the bottom of the ladder, the least restrictive measure is, of course, no intervention. As explained, this would be unacceptable because vaccination prevents serious risks to patients in healthcare facilities. Persuasion and nudging (in the form of opt-out systems) represent the next rungs up in the ladder, as they seek to influence behaviour and attitudes without depriving individuals of free choice and, according to some accounts, do not compromise autonomy (although this is debated with regard to nudging). Further up the ladder, incentives and disincentives are considered more coercive because they provide people with stronger reasons to do something that they might otherwise prefer not to do. Incentives would likely exert some influence on individuals’ decision-making, but it is at least debatable that they are coercive and, on some plausible accounts, they are not.^43^ Disincentives are more clearly coercive and the degree of coercion increases with the size of the disincentive. At the top of the ladder, penalties and force are the most coercive interventions because they leave individuals with few options and limited, if any, free choice. This type of intervention ladder has been applied to vaccination policies for the general population,^44^ but not specifically for FHCWs. So, in table 1, we propose an intervention ladder for vaccination policies for FHCWs.

**Table 1:** ’Intervention ladder’ adapted for mandatory vaccination of FHCWs from most to least coercive

Policy	Consequences of vaccine refusal*
Forced vaccination	Forcible vaccination using chemical or physical restraint, if required.
Compulsion/penalties	Fines or imprisonment; termination of employment; cancellation of professional registration.
Professional restrictions/conditions	Employment suspended; enforced leave; loss of salary for days not worked; admitting rights suspended; conditions imposed on professional registration preventing front-line healthcare work.
Redeployment	Redeployment to non-clinical duties, working from home. Restriction on direct clinical work with elderly, vulnerable, immunocompromised patients.
Loss of incentives	No access to employee privileges, such as additional paid leave; no access to restricted areas of the health service such as tea rooms or health club; no professional registration fee discount.
Nudging/libertarian paternalism	Opt-out policies, such as requiring FHCWs to sign a declinature statement explaining why they are refusing COVID-19 vaccination; reporting vaccination rates across different teams and highlighting underperforming teams to increase rates, making it harder for underperforming teams to say it is not possible to increase rates.
Persuasion	Education campaigns or professional development activities offered (but not mandated) to persuade FHCWs to reconsider.
No intervention	No intervention if FHCW refuses or declines vaccination.

*Does not apply to exemption on approved medical grounds.

FHCW, front-line healthcare worker.

According to this approach, despite the seriousness of COVID-19, coercive vaccination policies would only be required if FHCWs refuse vaccination and persuasion or nudging is ineffective. Given the need for immunity among FHCWs to be achieved rapidly once a vaccine is released, if we adopt this approach healthcare services need to start planning and communicating their proposed vaccination policies early, so that FHCWs objecting to vaccination can be targeted with education campaigns and given advance notice of the consequences of vaccine refusal. Racial, ethnic and other minority groups must receive messaging that addresses particular cultural or religious concerns. In spite of this, persuasion and nudging may be ineffective if the goal is to rapidly achieve close to 100% vaccination rates. In the case of influenza vaccination, it has been shown that policies requiring declinature statements are far less effective in increasing influenza vaccination rates in institutions aiming to achieve very high vaccination rates.^45^

The principle of least restrictive alternative, although very intuitive, is not without shortcomings. The most obvious is that it does not address the issue of probability of success and of risks. Less restrictive policies are preferable to more restrictive ones if they are both successful. However, if less restrictive policies are less likely to be successful (as is the case with influenza vaccination policies for FHCWs), then it means that by applying the principle we are assuming risk. If the success of the policy in question can prevent great harm—as seems to be the case for COVID-19 vaccination policies—then the risk of failing to achieve or delaying achieving the desired goal might be not worth taking.

If we want to abide by a principle of least restrictive alternative but, at the same time, minimise the risks of lower uptake and of infections in healthcare settings, we could consider a ‘conditional’ mandate in the first instance that preserves some liberty to refuse vaccination. Contact between unvaccinated FHCWs and vulnerable patients and colleagues could be restricted by redeploying FHCWs to non-clinical administrative duties or telehealth services, assuming their role can be fulfilled by other FHCWs who are vaccinated without significant costs to colleagues and to healthcare systems. This is less restrictive than exclusion from full employment or professional activity.

If redeployment to other roles is not possible, then unvaccinated FHCWs can be asked to take paid or unpaid leave. If, after that period of leave, the FHCW has not been vaccinated, then their employment or professional registration could be suspended or cancelled. Admittedly, this solution could impose significant financial costs on public health services. It could also entail significant professional burdens on vaccinated FHCWs who would be required to assume their unvaccinated colleagues’ clinical duties. The question then becomes whether, and to what extent, it is acceptable to impose these collective costs in order to accommodate FHCWs’ opposition to vaccines. Some small costs might be a reasonable price to pay, as long as safe and effective healthcare delivery is not compromised. However, it is important to bear in mind that the primary aim of healthcare systems and healthcare professions is providing adequate care to patients, and healthcare professionals’ personal freedom may and should be constrained accordingly.

Another possible concern with an overly restrictive policy is that it might result in significant workforce understaffing if too many FHCWs refuse vaccination. However, we consider this to be unlikely. Large health service organisations of between 5000 and 40 000 staff that adopt mandatory influenza vaccination policies have reported suspending or terminating the employment of no more than eight FHCWs.^46^ Moreover, healthcare services have a general duty to protect the occupational health of their staff.^47^ COVID-19 vaccines might entail more risks than influenza vaccines, but to assess whether it is justifiable to impose such risk on FHCWs through coercive public health policies, we should rely on objective standards of risk assessment, not on FHCWs’ subjective perceptions of risk.^29^ Thus, if the risk posed by COVID-19 vaccines is deemed acceptable by some objective standard (such as the one adopted by the authorities that license vaccines), then the risks of vaccination do not defeat mandatory measures. If, instead, the risk is considered high, then mandatory measures might have to be replaced by incentive schemes or supplemented by compensation-for-risk policies, which is a general point that has been made with regard to other forms of risk taking by FHCWs during the pandemic.^48^

Many health services already enforce infection control policies. FHCWs found breaching hand hygiene policies may be disciplined, suspended or reported to their professional registration board. Boards may deem such behaviour, if proven, to constitute unprofessional conduct and may reprimand or suspend an FHCW’s registration. Many health services already mandate influenza vaccination for FHCWs. Failure to be vaccinated can result in suspension or even termination of employment. Such action reduces vaccine refusal among FHCWs and improves patient safety.^49^ FHCWs who refuse vaccination, other than for genuine and compelling medical reasons, already face the choice of ‘no jab, no work’. This might involve annual vaccination certification as a requirement for continued employment, which could be monitored in the same way as other workplace vaccinations and infection control practices. This type of ‘fitness-for-duty’ policy already exists in some US hospitals.^50^

Julian Savulescu recently argued that in the case of a novel vaccine about which we have limited long-term safety data, the most appropriate way to incentivise community-wide vaccination might be to compensate individuals for the risk they incur.^51^ He argues that such a policy avoids coercion and increases peoples’ choices. In healthcare settings, incentives might include free meals, professional registration, parking and bonuses. However, unless these incentives are offered to all FHCWs, they may paradoxically discourage many FHCWs from voluntary vaccination if they see colleagues who refused vaccination receiving financial benefits. In addition, incentives may be costly and may be ineffective in persuading those with strong religious or moral convictions against vaccination.

## Defending conditional vaccination

We have argued for a conditional COVID-19 vaccination policy of FHCWs. One possible objection is that FHCWs’ own judgements, moral integrity or personal freedom ought to be prioritised. For instance, some FHCWs may be sceptical about the evidence of safety or effectiveness for a novel vaccine and may prefer to delay or defer vaccination. Some may be opposed on religious grounds. Libertarians might argue that individuals have the right to refuse acts that contravene their beliefs. The right of competent adults to refuse medical treatment, including vaccination, is a well-established ethical principle and is vigorously protected in most common law jurisdictions.

Moral integrity and authenticity require individuals to be faithful to their beliefs. When an individual acts against those beliefs, their identity may be injured. After all, the debate on conscientious objection in healthcare revolves precisely around this point. FHCWs may conscientiously object to providing contraception, pregnancy terminations or assistance with voluntary dying.^52 53^ In the same way, they may conscientiously object to vaccination.^54^ In fact, FHCWs refusing vaccination might argue that, unlike conscientious objection to abortion or assisted dying, they are refusing to treat themselves instead of refusing to treat their patients. In fact, it has even been argued that the right to conscientious objection in healthcare protects individuals and society^55^ because FHCWs committed to upholding moral ideals are resisting moral compromises that might otherwise lead to unethical or unprofessional conduct.^56^ However, leaving aside the complexities of the debate around conscientious objection in healthcare, these arguments as applied to vaccine refusal among FHCWs fail for a number of reasons.

First, the right to exercise free choice is not absolute. According to John Stuart Mill, personal freedoms extend only so far as they do not infringe on the legitimate interests of others.^57^ The European Convention on Human Rights also recognises that freedom of thought, conscience and religion may be limited by public safety concerns.^58^ The British Medical Association’s guidance on conscientious objection, for instance, recognises that, where a conflict arises between the interests of a patient and a doctor’s freedom to conscientiously object, the conflict should be resolved in favour of the patient.^59^ This is consistent with our argument.

Second, and more critically, FHCWs owe a professional and ethical obligation of non-maleficence towards their patients. One of the foundational principles of healthcare ethics is to ‘First Do No Harm’.^60^ Failing to avoid infecting others is ethically tantamount to harm through omission.^61^ Vaccine refusal puts patients at risk of infection and death. Given the evidence that vaccination prevents disease transmission to vulnerable patients and maintains the health of FHCWs, vaccination should be seen as a fundamental moral requirement for all FHCWs. The duty not to infect patients must take priority over any right to vaccine refusal. Indeed, most defenders of conscientious objection in healthcare concede that the duty to guarantee patients’ access to healthcare takes priority over FHCWs’ conscientious objection to practices like abortion.^62^ In our view, the same applies to vaccination.

Consider a case in which one individual risks sexually transmitting HIV to another individual by not wearing a condom. There is of course no obligation not to contract COVID-19 (or HIV), though there might be an obligation to minimise the risk of contracting it. In both cases, there is an unequivocal duty not to infect another person when this can be easily prevented, either by using condoms or being vaccinated. Vaccination is sufficiently easy and costless for most FHCWs that a positive duty to be vaccinated against COVID-19 could be as compelling as negative duties to avoid infecting patients with COVID-19. Since vaccination entails a very small cost to most FHCWs and a very large benefit to patients, FHCWs owe their patients a duty of care or even a *duty of easy rescue* 63 to be vaccinated.

Third, promoting the autonomy of FHCWs should not confine the autonomy of patients. Few patients can choose their FHCW. While mandatory vaccination policies are coercive for FHCWs, not instituting mandatory vaccination may be more coercive for patients. Whereas FHCWs can choose between vaccination and their job, patients cannot choose whether or not to get sick and cannot choose who cares for them when sick. They might have no alternative but be treated and cared for by unvaccinated FHCW. Therefore, the restriction on choice experienced by sick patients if no mandatory vaccination is imposed on FHCWs could be greater than the restriction experienced by FHCWs if such a policy is imposed.

Fourth, mandatory vaccination is arguably less burdensome than other mandatory infection control practices, as previously described. Employers and medical regulators already mandate infection control procedures on FHCWs that are restrictive and cause difficulty breathing, physical pain and skin breakdown. In addition, many health practitioner licensing boards already require FHCWs to be regularly tested for bloodborne viral infections, in recognition of their professional responsibility to prevent transmission to their patients.^64^ Requiring FHCWs to be vaccinated against COVID-19 is entirely consistent with established professional codes of conduct and ethics in many jurisdictions.

Fifth, there are concerns that the COVID-19 pandemic has revived antivaccine sentiments.^65^ Surveys show that public willingness to be vaccinated against COVID-19 varies from 49% in the USA,^66^ 62% in France, 80% in Denmark,^67^ 85% in Australia,^68^ to 93% in Indonesia.^69^ Willingness also varies according to race and ethnicity.^70^ The right of FHCWs to act on their personal views about COVID-19 vaccines must be balanced against the adverse consequences of their personal decisions on public health messaging. Hence, another powerful reason to mandate COVID-19 vaccination of FHCWs is to dispel public mistrust of vaccination and to encourage FHCWs to lead by example. Public trust in the healthcare system might diminish if it became known that FHCWs are forgoing vaccination and failing to prevent their patients getting sick or dying.^71^

Finally, every effort should be made to ensure that public health messaging about the benefits and risks of COVID-19 vaccination reaches disadvantaged groups, especially FHCWs from racial and ethnic minority groups that are over-represented in front-line healthcare roles and disproportionately impacted by serious adverse COVID-19 health outcomes. This benefits racially and ethnically diverse FHCWs themselves, and it assists them to spread important public health messages within their own culturally and linguistically diverse communities to ensure that coercive vaccination policies do not unfairly target and further disadvantage already marginalised groups.

## Conclusion

We need to consider how COVID-19 vaccines should be ethically distributed and what to do if people refuse to be vaccinated, given the impact that individual vaccine decisions have on society and specific communities. Within healthcare settings, vaccine choices can have even greater ramifications which, when coupled with the seriousness of COVID-19, justifies at least a mild form of mandatory vaccination policy for FHCWs. We believe that this should take the form of conditional employment or conditional professional registration, although temporary redeployment could be adopted if this does not entail significant costs to patients, to vaccinated colleagues and to the healthcare system. It is plausible that this would be the least restrictive policy that is most likely to achieve adequate vaccination uptake to reduce transmission of COVID-19 from unvaccinated FHCWs to their patients and colleagues and satisfy ethical and professional requirements. The risk to patients and the community posed by unvaccinated FHCWs outweighs concerns that conditional vaccination policies are coercive, provided that public health messages engage with people from diverse communities and groups within society.

## Data Availability

There are no data in this work.
